# A longitudinal linkage study of occupation and ischaemic heart disease in the general and Māori populations of New Zealand

**DOI:** 10.1371/journal.pone.0262636

**Published:** 2022-01-21

**Authors:** Lucy A. Barnes, Amanda Eng, Marine Corbin, Hayley J. Denison, Andrea t’ Mannetje, Stephen Haslett, Dave McLean, Lis Ellison-Loschmann, Rod Jackson, Jeroen Douwes

**Affiliations:** 1 Centre for Public Health Research, Massey University, Wellington, New Zealand; 2 School of Fundamental Sciences–Statistics, College of Sciences, Massey University, Palmerston North, New Zealand; 3 Statistical Consulting Unit, The Australian National University, Acton Australian Capital Territory, Canberra, Australia; 4 Faculty of Engineering and Information Sciences, University of Wollongong, New South Wales, Australia; 5 Health Services Research Centre, Victoria University of Wellington, Wellington, New Zealand; 6 Section of Epidemiology and Biostatistics, School of Population Health, Faculty of Medical and Health Sciences, The University of Auckland, Auckland, New Zealand; University of Hong Kong, HONG KONG

## Abstract

**Objectives:**

Occupation is a poorly characterised risk factor for cardiovascular disease (CVD) with females and indigenous populations under-represented in most research. This study assessed associations between occupation and ischaemic heart disease (IHD) in males and females of the general and Māori (indigenous people of NZ) populations of New Zealand (NZ).

**Methods:**

Two surveys of the NZ adult population (NZ Workforce Survey (NZWS); 2004–2006; n = 3003) and of the Māori population (NZWS Māori; 2009–2010; n = 2107) with detailed occupational histories were linked with routinely collected health data and followed-up until December 2018. Cox regression was used to calculate hazard ratios (HR) for IHD and “ever-worked” in any of the nine major occupational groups or 17 industries. Analyses were controlled for age, deprivation and smoking, and stratified by sex and survey.

**Results:**

‘Plant/machine operators and assemblers’ and ‘elementary occupations’ were positively associated with IHD in female Māori (HR 2.2, 95%CI 1.2–4.1 and HR 2.0, 1.1–3.8, respectively) and among NZWS males who had been employed as ‘plant/machine operators and assemblers’ for 10+ years (HR 1.7, 1.2–2.8). Working in the ‘manufacturing’ industry was also associated with IHD in NZWS females (HR 1.9, 1.1–3.7), whilst inverse associations were observed for ‘technicians and associate professionals’ (HR 0.5, 0.3–0.8) in NZWS males. For ‘clerks’, a positive association was found for NZWS males (HR 1.8, 1.2–2.7), whilst an inverse association was observed for Māori females (HR 0.4, 0.2–0.8).

**Conclusion:**

Associations with IHD differed significantly across occupational groups and were not consistent across males and females or for Māori and the general population, even within the same occupational groups, suggesting that current knowledge regarding the association between occupation and IHD may not be generalisable across different population groups.

## Introduction

Cardiovascular disease (CVD) is a leading cause of death, with ischaemic heart disease (IHD) making up the largest proportion [1]. Risk factors include smoking, lack of physical activity, overweight, high blood pressure, high cholesterol and diabetes mellitus, as well as non-modifiable characteristics such as age, sex, ethnicity and family history [2].

Occupation is another modifiable risk factor, but the evidence is limited [3]. In New Zealand (NZ), 19% of IHD mortality may be attributable to occupational risk factors for males and 9% for females [4]. The first NZ study to examine specific occupations and IHD mortality identified elevated risks in spinners, weavers and dryers, and shoemakers and leather good workers, but the study was conducted more than three decades ago and only included males [5]. A later update for the period 2001–2005 identified plant/machine operators and assemblers as having high rates of IHD mortality for NZ males [6]. International research has highlighted drivers, police officers, factory workers, and blue-collar occupations more generally, to be among high-risk occupations [7, 8].

Previous research on occupational risk factors had some limitations: many cohort studies were based on death records, which did not include full occupational histories and were not controlled for important confounders (e.g. smoking) [5, 6], while cross-sectional studies relied on self-reported IHD [8]. Furthermore, despite sex-specific differences in CVD risk [9] and occupational exposure profiles [10], previous research has predominantly focused on male-dominated occupations or only included men. Similarly, little research has been conducted in ethnic minority groups, despite evidence suggesting differences in cardiovascular risk between ethnicities [11]. In NZ, Maori (the indigenous people of NZ) have a considerably greater CVD mortality compared to NZ Europeans [12], and occupational exposure patterns are also significantly different [13], hence occupational risk factors may not be the same between both groups. Therefore, effective workplace interventions may need to take into account sex and ethnic differences.

In this study we used detailed occupational history and lifestyle information previously collected as part of two NZ Workforce Surveys: one in Māori [13] who make up ~16.5% of the population [14] and one in the general population [15] comprising 70.2% of European origin, 8.1% Pacific and 15.1% Asian peoples. Data was linked to routinely recorded health information to identify incident IHD and assess the association with occupation for males and females in the Māori and general populations. Although some information on specific self-reported occupational exposures was available, this has been reported in a separate publication [16] as this is not the focus of the current study.

## Methods

This is a longitudinal study using occupational history and lifestyle information from two previous occupational surveys. Participants were followed-up for 7–14 years from date of interview, for incident IHD using linked administrative health data.

### Workforce surveys

The methods for the New Zealand Workforce Surveys (NZWS [15] and Māori NZWS [13]) have been described in detail previously. Briefly, a random proportionally stratified, systematic and self-weighted sample of people aged 20–64 years was selected from the Māori and general electoral rolls. For the NZWS, participants (including NZ European, Māori, Pasifika and other ethnic groups) were interviewed by phone from 2004–2006. In total, 3003 participants (37%) completed the survey [15]. The Māori NZWS was conducted from 2009–2010 using the same methodology and resulted in 2107 participants (29%) completing the survey [13]. Two participants were included in both surveys and we therefore excluded their most recent interview (i.e. the Māori NZWS).

The questionnaire included questions about lifetime work history, current workplace exposures, and demographic and lifestyle factors. Ethics approval was granted by the Massey University Human Ethics Committee (NZWS–WGTN 03/133, Māori NZWS–MUHEC 08/28) and from the New Zealand Health and Disability Ethics Committee for the linkage of the two surveys (16/NTB/173).

As previously described, potential response bias from low survey response was small [17]. While some groups were under-represented including, younger ages, higher deprivation, and Māori (in the NZWS), the prevalence of key survey variables (both occupational exposure and health related variables) were unchanged after standardising to the demographic distribution of the source population, and similar between early and late responders.

### CVD risk factors assessed via questionnaire

Start date, end date and duration for each job were recorded, and jobs of ≥6 months were coded using the NZ Standard Classification of Occupations (NZSCO) 1999, which is a hierarchical skills-based classification with nine major occupational group [18]. Work histories also included information about the main activity of the organisation for each job, which was coded for industry using the Australian and New Zealand Standard Industrial Classification (ANZSIC) 1996 [19]. Age at interview was categorised: 20–34, 35–44, 45–54 and 55+ years. Socioeconomic status (SES) was assessed using the NZ Deprivation Index 2006 (NZDep2006), a census-based index with a relative deprivation score ranging from 1–10, based on place of residence. The distribution of deprivation is presented in quintiles, but for subsequent analyses it was dichotomised, combining scores 1–8 (least deprived) and 9–10 (most deprived).

Smoking was analysed as never/ever and as pack-years, calculated from the number of cigarettes smoked per day divided by 20, multiplied by the number of years smoked. Body Mass Index (BMI) was calculated using self-reported height and weight grouped into four categories (i.e. <18.5, 18.5–24.9, 25–29.9, 30+) [20].

### IHD events identified from linked health data

IHD events were ascertained by linking de-identified survey information to the Integrated Data Infrastructure (IDI), a longitudinal meta-dataset linked at the individual level, consisting of data from government agencies [21]. Before linkage, probabilistic matching was conducted to determine participants’ National Health Index (NHI), which resulted in 98% of respondents being successfully matched and linkable [22]. Ministry of Health records including mortality, public hospital diagnoses, and pharmaceutical dispensing were accessed for all participants.

IHD was identified using a previously developed definition [23]: it includes IHD deaths, hospital diagnoses and procedures using International Classification of Disease (ICD) codes and dispensings of anti-anginal drugs recorded in the pharmaceutical claims dataset (Table 1). Primary health care information was not used as it is not available in the IDI.

**Table 1:** IHD definition.

IDI Data Source	ICD Code or Criteria	Details
Mortality data (1988–31 Dec 2016)	ICD-9-CMA 410, ICD-10-AM I21	Acute myocardial infarction
	ICD-10-AM I22	Subsequent myocardial infarction
	ICD-9-CMA 411, ICD-10-AM I24	Other acute and subacute forms of ischaemic heart disease
	ICD-9-CMA I23	Certain current complications following myocardial infarction (within the 28-day period)
National Minimum Dataset (NMDS): Publicly funded hospital discharges–diagnosis/procedure information (1988–31 Dec 2018)	ICD-9-CMA 413, ICD-10-AM I20	Angina pectoris
	ICD-9-CMA 412, ICD-9-CMA 414, ICD-10-AM I25*	Chronic ischaemic heart disease
	ICD-9-CMA V45.81	Aortocoronary bypass status
	ICD-9-CMA V45.82	Percutaneous transluminal coronary angioplasty status
	ICD-10-AM Z95.1	Presence of aortocoronary bypass graft
	ICD-10-AM Z95.5	Presence of coronary angioplasty implant and graft
	ICD-9- CMA 36.0x, 36.1x	Procedures
	ICD-10-AM 3530400, 3530401, 3530500, 3530501, 3531000, 3531001, 3531002, 3531003, 3531004, 3531005, 3849700, 3849701, 3849702, 3849703, 3849704, 03849705, 3849706, 3849707, 3850000, 3850001, 3850002, 3850003, 3850004, 3850300, 3850301, 3850302, 3850303, 3850304, 9020100, 9020101, 9020102, 9020103, 3863700, 3845619, 3865308, 3850500	
Pharmaceutical Data (2005–31 Dec 2018)	1577 Glyceryl trinitrate	≥2 dispensings of any of these medications within 12 months
	2377 Isosorbide dinitrate	
	2836 Isosorbide mononitrate	
	1292 Nicorandil	
	1949 Perhexiline maleate	

*ICD-10-AM I25.2 was excluded as it refers to old myocardial infarction and therefore, not an incident IHD event

### Follow up of IHD events

Participants were followed from the date of interview: 2004–2006 for the general population and 2009–2011 for Māori. Participants with prior IHD were excluded. Participants who had moved overseas or died were identified through immigration and mortality data, respectively, and were censored from that time point. The end of follow-up was 31 December 2018; this is the date last observed for those that were not lost to follow-up, not deceased, or did not have an IHD event.

### Statistical analyses

Cox proportional hazards regression was used to estimate hazard ratios (HR) of first IHD event for “ever worked” in a particular occupational group compared to “never worked” in that group, for each of the nine major NZSCO occupational groups and 17 ANZSIC industry groups. HRs were also estimated for the 23 two-digit NZSCO codes.

Work duration in each occupational group was categorised as never or <0.5, 0.5–2, >2–10, and >10 years. For 116 job records duration was missing and these were excluded from duration analyses. Duration was fitted as a categorical variable using the ‘never’ category as the reference group (only HRs for 10+ years are presented).

Analyses are presented as age-adjusted (with no further adjustments) and age (4 categories), SES (highest deprivation quintile vs all other), and smoking status (ever/never)-adjusted HRs. Additional analyses adjusting for pack-years and BMI were also conducted (S1 Table). For the NSWS population we also conducted analyses with further adjustment for ethnicity, but as results did not change, and the number of subjects for some ethnic groups were very small (with only few IHD cases in each group), these are not presented.

In compliance with the IDI confidentiality requirements, all frequencies were rounded to the nearest multiple of three and percentages were calculated from the rounded counts (hence the number of total participants in each table varying slightly, and percentages not adding up to exactly 100%). All statistical tests were performed on unrounded counts. All counts under six and the HRs derived from these are suppressed according to IDI confidentiality requirements (marked as ‘S’ in the tables).

The same participants and data linkage methods have been used in subsequent analyses to assess the association between a range of occupational exposures (e.g. dust, stress and sitting) and incident IHD [16].

## Results

The average follow-up for the NZWS and Māori NZWS survey was 12.1 and 7.5 years, respectively. For 85 participants (45 NZWS and 40 Māori NZWS) we were not able to link survey and health data, and 213 participants had had an IHD event prior to interview (83 NZWS and 130 Māori NZWS), leaving a total of 2874 participants in the NZWS survey and 1935 in the Māori NZWS survey for analyses.

### Incident IHD

The percentage of incident IHD cases identified through hospital discharges ranged from 73–80% across males and females of both surveys, while 19–25% of cases were identified through pharmaceutical dispensings only; mortality records identified the remainder. In the NZWS survey, 99 males and 36 females had a first IHD event during follow-up; in the Māori survey, 51 males and 42 females had a first IHD event. Males had proportionally more incident IHD in both surveys (Table 2). Incident IHD cases were more likely to be aged 55+ years, to have ever smoked, be deprived, and be obese (Table 2). Obesity, smoking and deprivation were more common in the Māori than the general NZWS survey. These risk factors were also most prevalent in males, but differences were less pronounced in the Māori NZWS.

**Table 2:** Population characteristics.

	NZWS Survey				Māori NZWS Survey			
	Total n = 2874		IHD cases n = 135		Total n = 1935		IHD cases n = 93	
	Males	Females	Males	Females	Males	Females	Males	Females
	1350 (47.0%)	1524 (53.0%)	99 (73.3%)	36 (26.6%)	852 (44.0%)	1083 (56.0%)	51 (54.8%)	42 (45.2%)
	n (%)	n (%)	n (%)	n (%)	n	n (%)	n (%)	n (%)
**Age at interview**								
20–34	279 (20.7)	333 (21.9)	S	S	159 (18.7)	207 (19.1)	S	S
35–44	324 (24.0)	459 (30.1)	15 (15.2)	6 (16.7)	201 (23.6)	255 (23.5)	S	S
45–54	390 (28.9)	462 (30.3)	30 (30.3)	15 (41.7)	210 (24.6)	273 (25.2)	9 (17.6)	12 (28.6)
55+	354 (26.2)	273 (17.9)	54 (54.5)	15 (41.7)	288 (33.8)	348 (32.1)	39 (76.5)	27 (64.3)
**Interview year**								
2004	147 (10.9)	198 (13.0)	15 (15.2)	S	N/A	N/A	N/A	N/A
2005	678 (50.2)	738 (48.4)	57 (57.6)	24 (66.7)	N/A	N/A	N/A	N/A
2006	528 (39.1)	588 (38.6)	30 (30.3)	9 (25.0)	N/A	N/A	N/A	N/A
2007	S	S			N/A	N/A	N/A	N/A
2009	N/A	N/A	N/A	N/A	156 (18.3)	207 (19.1)	12 (23.5)	12 (28.6)
2010	N/A	N/A	N/A	N/A	456 (53.5)	585 (54.0)	30 (58.8)	21 (50.0)
2011	N/A	N/A	N/A	N/A	240 (28.2)	288 (26.6)	12 (23.5)	9 (21.4)
**Ethnicity**								
Pākehā	1080 (80.4)	1206 (79.1)	78 (78.8)	33 (91.7)				
Māori^a^	99 (7.4)	156 (10.2)	12 (12.1)	S	852 (100.0)	1083 (100.0)	51 (100.0)	42 (100.0)
Pacific peoples	21 (1.6)	33 (2.1)	S	S				
Other	144 (10.7)	129 (8.5)	9 (9.1)	S				
*Missing*	S							
**Smoking**								
Ever	681 (50.6)	738 (48.4)	60 (60.6)	21 (58.3)	513 (60.4)	717 (66.4)	30 (58.8)	30 (71.4)
Current	246 (18.2)	282 (18.5)	27 (27.3)	9 (25.0)	210 (24.6)	327 (30.2)	6 (11.8)	12 (28.6)
*Missing*	S	S			S	S		
**Body Mass Index**								
<18.5	S	27 (1.9)	S	S	S	18 (1.9)	S	S
18.5–24.9	465 (35.1)	717 (49.7)	2 (28.1)	12 (36.4)	135 (16.4)	297 (30.8)	S	9 (23.1)
25–29.9	606 (45.7)	429 (29.7)	39 (40.6)	12 (36.4)	318 (38.7)	255 (26.5)	12 (24.5)	12 (30.8)
30+	249 (18.8)	267 (18.5)	30 (31.3)	9 (27.3)	366 (44.5)	387 (40.2)	30 (61.2)	18 (46.2)
*Missing*	21	84	S	S	30	120	S	S
**Deprivation Index 2006**								
1–2 (least deprived)	390 (28.9)	378 (24.8)	24 (24.2)	9 (25.0)	129 (15.2)	138 (12.7)	S	S
3–4	306 (22.7)	324 (21.3)	21 (21.2)	6 (16.7)	135 (15.9)	156 (14.4)	6 (11.8)	S
5–6	273 (20.2)	354 (23.2)	18 (18.2)	6 (16.7)	147 (17.3)	195 (18.0)	9 (17.6)	S
7–8	222 (16.4)	279 (18.3)	12 (12.1)	9 (25.0)	201 (23.7)	246 (22.7)	12 (23.5)	18 (42.9)
9–10 (most deprived)	162 (12.0)	186 (12.2)	18 (18.2)	6 (16.7)	234 (27.6)	351 (32.4)	21 (41.2)	15 (35.7)
*Missing*		S			S	S		

Following IDI protocols, frequencies have been rounded to the nearest multiple of three and percentages calculated from those rounded counts. (S = suppressed)

^a^Indigenous people of New Zealand

### Occupation and industry and IHD

Among NZWS males, an inverse association with IHD was observed for technicians and associate professionals (Table 3; HR 0.5 (0.3–0.8)) whereas clerks had a greater risk (HR 1.8 (1.2–2.7)). Associations were less pronounced for those who worked 10+ years in these occupations (HR 0.8 (0.8–1.0) and 1.6 (0.8–3.0), respectively; Table 3). Employment for 10+ years as a plant/machine operator and assembler was also positively associated with IHD (HR 1.7 (1.2–2.8)).

**Table 3:** Associations between occupational groups and IHD.

Occupational Group	Total (n)	IHD cases (n)	HR (95%CI)^a^	HR (95%CI)^b^	Total (n)	IHD cases (n)	HR (95%CI)^a^	HR (95%CI)^b^
**NZWS**	**Males**				**Females**			
1. Legislators, Admin. & Managers (ever)	435	33	0.9 (0.6–1.4)	0.9 (0.6–1.4)	333	9	1.0 (0.5–2.2)	1.0 (0.5–2.2)
(employed 10+ years)	198	12	0.6 (0.4–1.2)	0.7 (0.4–1.3)	69	S	S	S
2. Professionals (ever)	399	24	0.7 (0.4–1.0)	0.7 (0.4–1.1)	591	18	1.0 (0.5–2.0)	1.1 (0.6–2.1)
(employed 10+ years)	180	12	0.6 (0.3–1.1)	0.9 (0.7–1.1)	249	6	0.7 (0.3–1.8)	0.8 (0.3–1.9)
3. Technicians & Assoc. Professionals (ever)	447	21	0.5 (0.3–0.8)**	0.5 (0.3–0.8)**	633	12	0.7 (0.4–1.4)	0.7 (0.4–1.5)
(employed 10+ years)	147	12	0.7 (0.4–1.3)	0.8 (0.7–1.0)*	159	S	S	S
4. Clerks (ever)	285	33	1.7 (1.1–2.6)**	1.8 (1.2–2.7)**	822	21	1.0 (0.5–2.0)	1.0 (0.5–1.9)
(employed 10+ years)	72	12	1.5 (0.8–3.0)	1.6 (0.8–3.0)	255	9	1.1 (0.5–2.6)	1.1 (0.5–2.6)
5. Service & Sales Workers (ever)	369	18	0.6 (0.6–1.1)	0.6 (0.4–1.0)	750	21	1.6 (0.8–3.2)	1.5 (0.8–3.0)
(employed 10+ years)	81	6	0.8 (0.3–1.8)	0.8 (0.3–1.8)	165	9	2.0 (0.8–4.9)	1.9 (0.8–4.6)
6. Agriculture & Fishery Workers (ever)	315	24	1.0 (0.6–1.5)	0.9 (0.6–1.5)	183	6	1.2 (0.5–2.9)	1.2 (0.5–2.8)
(employed 10+ years)	120	9	0.8 (0.4–1.6)	0.8 (0.4–1.6)	42	S	S	S
7. Trades Workers (ever)	459	36	1.1 (0.8–1.7)	1.1 (0.7–1.6)	60	S	S	S
(employed 10+ years)	246	24	1.2 (0.7–1.9)	1.1 (0.7–1.8)	18	S	S	S
8. Plant/Machine Operators & Assemblers (ever)	393	36	1.3 (0.9–2.0)	1.2 (0.8–1.8)	198	6	1.3 (0.6–3.1)	1.2 (0.5–2.8)
(employed 10+ years)	141	21	1.9 (1.2–3.2)**	1.7 (1.2–2.8)*	33	S	S	S
9. Elementary Occupations (ever)	327	27	1.3 (0.8–2.1)	1.2 (0.8–1.9)	237	6	1.2 (0.5–2.6)	1.1 (0.5–2.5)
(employed 10+ years)	33	S	s	S	33	S	S	S
**Māori NZWS**	**Males**				**Females**			
1. Legislators, Admin. & Managers (ever)	201	18	1.2 (0.7–2.2)	1.3 (0.7–2.3)	264	S	S	S
(employed 10+ years)	81	9	1.0 (0.5–2.2)	1.1 (0.5–2.4)	66	S	S	S
2. Professionals (ever)	180	9	0.8 (0.4–1.6)	0.8 (0.4–1.6)	363	9	0.7 (0.3–1.4)	0.7 (0.3–1.4)
(employed 10+ years)	69	6	1.1 (0.5–2.4)	1.1 (0.5–2.6)	120	6	0.8 (0.3–1.9)	0.8 (0.3–1.9)
3. Technicians & Assoc. Professionals (ever)	234	12	0.7 (0.4–1.4)	0.7 (0.4–1.4)	402	9	0.5 (0.3–1.1)	0.5 (0.3–1.1)
(employed 10+ years)	60	S	S	S	96	S	S	S
4. Clerks (ever)	174	9	0.8 (0.4–1.4)	0.8 (0.4–1.7)	543	12	0.4 (0.2–0.8)**	0.4 (0.2–0.8)*
(employed 10+ years)	33	S	S	S	144	S	S	S
5. Service & Sales Workers (ever)	270	15	1.0 (0.6–1.9)	1.0 (0.6–1.9)	651	27	1.5 (0.7–2.9)	1.4 (0.7–2.8)
(employed 10+ years)	63	6	1.2 (0.5–2.9)	1.3 (0.5–3.1)	153	S	S	S
6. Agriculture & Fishery Workers (ever)	297	24	1.3 (0.7–2.2)	1.2 (0.7–2.2)	210	12	1.5 (0.8–3.0)	1.5 (0.8–3.0)
(employed 10+ years)	81	9	1.2 (0.5–2.5	1.1 (0.5–2.4)	48	S	S	S
7. Trades Workers (ever)	336	21	0.8 (0.4–1.4)	0.7 (0.4–1.3)	72	6	2.1 (0.9–4.9)	2.0 (0.8–4.8)
(employed 10+ years)	132	9	0.8 (0.4–1.6)	0.7 (0.3–1.5)	S	S	S	S
8. Plant/Machine Operators & Assemblers (ever)	435	33	1.4 (0.8–2.4)	1.3 (0.7–2.3)	312	24	2.2 (1.2–4.1)**	2.2 (1.2–4.1)*
(employed 10+ years)	204	21	1.5 (0.8–2.9)	1.5 (0.8–2.8)	54	S	S	S
9. Elementary Occupations (ever)	369	21	1.5 (0.8–2.6)	1.4 (0.8–2.4)	294	18	2.0 (1.1–3.8)**	2.0 (1.1–3.8)*
(employed 10+ years)	42	S	S	S	45	6	3.2 (1.37.9)**	3.2 (1.3–7.9)*

*P value <0.05

**P value <0.01.

Following IDI protocols, frequencies have been rounded to the nearest multiple of three and percentages calculated from those rounded counts. The hazard ratios and associated 95% confidence intervals are presented in their raw form and were calculated using the unrounded counts. (S = suppressed)

^a^Adjusted for age group

^b^Adjusted for age group, high deprivation and smoking status

For NZWS females, none of the main occupational groups showed statistically significant associations (Table 3). When considering more detailed 2-digit occupational groups (S2 Table), the “52- Salespersons, Demonstrators and Models” occupational group was associated with an increased risk (HR 2.2 (1.2–4.3)). An increased risk was also observed for ever employment in the manufacturing industry (S3 Table; HR 1.9 (1.1–3.7)).

For Māori males, none of the main occupational or industry groups showed statistically significant associations after adjustment for age, SES and smoking (Table 3 and S3 Table).

Among Māori females, clerks were less likely to have new-onset IHD (HR 0.4 (0.2–0.8), Table 3), in contrast with what was observed for NZWS males. Employment as plant/machine operators and assemblers or in elementary occupations was associated with an increased risk (HR 2.2 (1.2–4.1) and 2.0 (1.1–3.8), respectively) and 10+ years of employment in elementary occupations was associated with a three-times greater risk (HR 3.2 (1.3–7.9)). Employment in the agriculture, forestry and fishing industry for 10+ years was also associated with an increased risk (S3 Table; HR 2.5 (1.0–6.1)). Analyses involving <2 years and 2–10 years employment duration showed similar findings to the “ever worked” analyses (data not shown).

### Additional adjustments for potential confounders

Results reported above were adjusted for “ever/never having smoked” as “pack-years” had more missing information. Also, as 5% of participants had missing information on BMI we did not adjust the analyses for this. HRs adjusted for pack-years and BMI are presented in S1 Table, and compared with the unadjusted HRs, whilst restricting the sample to participants for whom we had complete data on pack-years and BMI to ensure comparisons were done on the same groups. These additional analyses did not change the results.

## Discussion

This study, which linked detailed occupational histories to IHD using routinely collected health data, showed increased IHD risks for several occupational groups, with some marked sex and ethnic differences.

Incident IHD was more common in several blue-collar occupational groups such as plant/machine operators and assemblers, and elementary workers, consistent with previous research showing increased IHD risk in blue-collar workers and NZ-specific data showing higher IHD mortality in plant/machine operators and assemblers [6, 8, 24]. Smoking and BMI are unlikely to explain this as analyses were adjusted for these factors. Blue-collar workers are often exposed to multiple hazards, including carbon monoxide, irregular schedules, noise, and vigorous physical activity, which have previously been associated (inconsistently) with CVD [25]. The increased risk for blue-collar occupations was most pronounced for Māori females, with employment in plant/machine operators and assemblers, and elementary workers doubling the risk of IHD. It is possible that within these broad occupational groups Māori women are more frequently involved in specific high-risk jobs, but this could not be confirmed. Alternatively, increased risk in women may be due to differential physiological responses to the same occupational exposures. Furthermore, as these occupations traditionally employ more males, it is possible that females are exposed to additional risk factors including: working with equipment not designed for females, social isolation, gender discrimination and sexual harassment [24]. In addition, as we have shown previously, Māori are more exposed to physical factors [13] and may also suffer racial discrimination within the workplace, potentially compounding the risk for female Māori [26].

A strong positive association with IHD was shown for clerks in male NZWS participants. An increased CVD risk has previously been reported for clerical workers, although this has primarily been found in females [27] and may be due to obesity and other CVD risk factors [28]. However, as we reported earlier [22], no differences in obesity, smoking, diabetes or high cholesterol were observed between male NZWS clerks and other occupational groups; nonetheless, male clerks in the NZWS survey had twice the risk of high blood pressure (females had 1.5 times the risk). Although evidence is inconsistent, some studies have linked sedentary behaviour, common among clerical workers, to increased blood pressure, which may explain the increase in CVD risk [29]. In addition, job strain among clerical workers, and associated with CVD [30], may also play a role.

Our finding of a reduced risk among female Māori clerks is opposite to what we found for men, and inconsistent with previous research (see above). When comparing specific job titles within the group of clerks, there were notable differences, with substantially more females working as ‘secretaries and keyboard operating clerks’, while a greater proportion of males worked as ‘material recording and transport clerks’. The occupational exposures associated with these sub-groups may be different and may explain the differential findings for male and female clerks. In addition, as we have shown previously, occupational exposures between Māori and non-Māori may differ, even within the same job title, with Māori involved in more physically demanding tasks [13].

Opposing results were also found for male and female service and sales workers in the general survey: NZWS females who worked as ‘52-Salespersons, Demonstrators and Models’ had an increased IHD risk, while NZWS males in the same occupational group had a reduced risk. Specific occupations, tasks and related exposures may differ between males and females working in this occupational group, such as sedentary work and standing at work, both of which have been (inconsistently) linked to IHD [31, 32].

Different results were observed for employment in agriculture, which was a risk factor for Māori females but not NZWS males. Previous research of agricultural workers has been inconsistent with some studies showing a high prevalence of multiple risk factors, such as high blood pressure and high cholesterol [33], while other studies have demonstrated favourable CVD risk factor profiles in this industry, possibly due to better access to nutritious foods and higher levels of physical activity [34]. Our study suggests that Māori females working in agriculture may not equally benefit from this, but the reasons for this are unclear.

Technicians and associate professionals were less likely to experience IHD and this was shown across all subgroups, although it only reached statistical significance in NZWS males. In general, white-collar occupations are less exposed to hazardous occupational exposures, and this may in part explain the protective effect. However, similar trends were not found for other non-manual workers (clerks, legislators, administrators and managers).

As previously stated, the same occupational surveys and linkage methods have been used to investigate potential causal occupational exposures that may be relevant in the associations between occupational group and IHD [16].

This study has several limitations. The number of IHD cases was relatively small, with insufficient power to focus on more detailed occupational groups. Also, as we studied 9 occupational groups and 17 industries across both sexes in two surveys, involving many comparisons, some of our results may be due to chance. In fact, 222 statistical tests were conducted, equating to an expected number of false positives of 2 and 11 at the p<0.01 levels p<0.05 level, respectively. A similar rider applies to supplementary tables with 195 further tests, 17 and 11 significant at p<0.05 and p<0.01, and expected false positives of 10 and 2, respectively. Therefore, results, particularly those not previously reported in the literature, should be interpreted with caution. In addition to type I error (false positive findings), type II error (false negative findings) may have occurred, particularly for analyses that were based on relatively small number of exposed workers. As a results, it cannot be excluded that non-significant findings could in fact be reflective of true associations.

There are other limitations including the variable date ranges of available health data (Table 1), which meant that the IHD definition varied somewhat across time. In particular, pharmaceutical data were only available from 2005, and mortality data with confirmed ICD codes only up to December 2016 (although non-coded death data was available until the end of December 2018). However, the impact of this is likely small, as the majority of case ascertainment was based on hospital discharges, which covered the whole follow-up period. In addition, the definition of IHD was based on healthcare use, which may be different between genders and ethnicities [35, 36]. As primary health care data and private hospitalisation information are not available in the IDI, IHD events that did not result in a public hospitalisation, specific pharmaceutical prescription, or death, were not included, potentially resulting in a small undercount of IHD cases. In contrast, the inclusion of angina pectoris in the definition of IHD may have led to an overestimation of IHD. In particular, a proportion of cases was defined only on drug dispensings for angina pectoris; this may have resulted in some subjects incorrectly being defined as an IHD case (a diagnosis based on only drug prescription is likely to result in some misclassification). The inclusion of angina pectoris also limits the comparability with some other studies that have primarily focused on more defined endpoints, such as myocardial infarction.

Residual confounding is another limitation to this study. We did not adjust the analyses for high blood pressure, high cholesterol or diabetes as it is likely that these conditions are on the causal pathway between exposure and IHD in which case it would be inappropriate to adjust for these factors. However, there is potential for other unmeasured confounding (e.g. diet, physical activity, family history of CVD and alcohol consumption) that we were not able to account for. There is also the potential of recall bias; however, this is unlikely to have played a role as participants had no knowledge of the specific exposure-IHD associations under investigation, as data on occupation was obtained from comprehensive lifetime work histories collected prior to the conception of the current study on IHD.

A strength of the study is its longitudinal design, which did not rely on self-reports of outcomes, therefore reducing the risk of bias. The occupational histories also allowed for assessment of duration of employment. A unique feature of this study is that it included >50% females and a large proportion of Māori, a population that has not previously been studied with respect to occupation and IHD.

In conclusion, the association between occupation and IHD differed across occupational groups, even after adjustment for known risk factors. Occupational risk patterns also differed between males and females, and between the Māori and general populations, suggesting that current knowledge regarding occupation and IHD may not be generalisable across all population groups.

## Supporting information

S1 Table Associations between occupational groups and IHD, excluding participants with missing BMI or pack-years information. (DOCX)

S2 Table Associations between NZSCO two-digit occupational groups and IHD. (DOCX)

S3 Table Associations between industry group and IHD. (DOCX)
