# Supplementary material for: A longitudinal linkage study of occupation and ischaemic heart disease in the general and Māori populations of New Zealand
Source: PLoS One. 2022 Jan 21;17(1):e0262636. doi: 10.1371/journal.pone.0262636 (PMC8782384; doi:10.1371/journal.pone.0262636)
Supplement: S1 Table — (DOCX) [file pone.0262636.s001.docx]

| **S1 Table: Associations between occupational groups and IHD, excluding participants with missing BMI or pack-years information^a^** | | | | | | | | | | |
| --- | --- | --- | --- | --- | --- | --- | --- | --- | --- | --- |
| **Occupational Group** | **Total**  **(n)** | **IHD cases**  **(n)** | **HR (95%CI)^a^** | **HR (95%CI)^c^** | **HR (95%CI)^d^** | **Total**  **(n)** | **IHD cases**  **(n)** | **HR (95%CI)^a^** | **HR (95%CI)^c^** | **HR (95%CI)^d^** |
| **NZWS** | **Males** | | | | | **Females** | | | | |
| 1. Legislators, Admin. and Managers (ever) | 432 | 33 | 0.9 (0.6-1.4) | 0.9 (0.6-1.4) | 0.9 (0.6-1.4) | 318 | 6 | 1.0 (0.4-2.1) | 1.0 (0.4-2.2) | 1.0 (0.4-2.1) |
| 2. Professionals (ever) | 390 | 21 | 0.7 (0.4-1.1) | 0.7 (0.4-1.1) | 0.7 (0.4-1.1) | 564 | 15 | 1.0 (0.5-2.0) | 1.0 (0.5-2.1) | 1.0 (0.5-2.0) |
| 3. Technicians & Assoc. Professionals (ever) | 444 | 21 | 0.5 (0.3-0.9)***** | 0.5 (0.3-0.9)****** | 0.5 (0.3-0.9)***** | 603 | 12 | 0.7 (0.4-1.5) | 0.7 (0.4-1.5) | 0.7 (0.4-1.5) |
| 4. Clerks (ever) | 282 | 30 | 1.8 (1.2-2.8)****** | 1.9 (1.2-2.9)****** | 1.9 (1.2-2.9)****** | 777 | 18 | 1.0 (0.5-1.9) | 1.0 (0.5-2.0) | 0.9 (0.5-1.9) |
| 5. Service & Sales Workers (ever) | 366 | 18 | 0.6 (0.4-1.0) | 0.6 (0.4-1.0) | 0.6 (0.3-1.0) | 708 | 21 | 1.4 (0.7-2.9) | 1.5 (0.7-2.9) | 1.5 (0.7-3.0) |
| 6. Agriculture & Fishery Workers (ever) | 309 | 27 | 1.0 (0.6-1.5) | 0.9 (0.6-1.4) | 0.9 (0.6-1.5) | 174 | S | S | S | S |
| 7. Trades Workers (ever) | 453 | 36 | 1.1 (0.7-1.6) | 1.1 (0.8-1.8) | 1.1 (0.7-1.6) | 60 | S | S | S | S |
| 8. Plant/Machine Operators & Assemblers (ever) | 384 | 36 | 1.2 (0.8-1.8) | 1.2 (0.8-1.8) | 1.1 (0.7-1.7) | 192 | 6 | 1.1 (0.5-2.7) | 1.1 (0.4-2.7) | 1.1 (0.5-2.8) |
| 9. Elementary Occupations (ever) | 321 | 27 | 1.2 (0.8-1.9) | 1.2 (0.8-1.9) | 1.2 (0.8-1.9) | 219 | 6 | 1.0 (0.4-2.4) | 1.0 (0.4-2.4) | 1.0 (0.4-2.4) |
| **Māori NZWS** | **Males** | | | | | **Females** | | | | |
| 1. Legislators, Admin. and Managers (ever) | 198 | 15 | 1.3 (0.7-2.3) | 1.4 (0.8-2.6) | 1.3 (0.7-2.5) | 252 | S | S | S | S |
| 2. Professionals (ever) | 177 | 9 | 0.7 (0.3-1.5) | 0.7 (0.4-1.5) | 0.8 (0.4-1.6) | 333 | 12 | 0.7 (0.4-1.5) | 0.7 (0.4-1.5) | 0.7 (0.4-1.5) |
| 3. Technicians & Assoc. Professionals (ever) | 228 | 12 | 0.8 (0.4—1.5) | 0.8 (0.4-1.5) | 0.8 (0.4-1.7) | 363 | 12 | 0.5 (0.3-1.1) | 0.5 (0.3-1.1) | 0.5 (0.3-1.1) |
| 4. Clerks (ever) | 174 | 9 | 0.9 (0.4-1.8) | 1.0 (0.5-2.0) | 1.0 (0.5-2.1) | 480 | 12 | 0.5 (0.2-0.9)***** | 0.5 (0.2-0.9)***** | 0.5 (0.2-0.9)***** |
| 5. Service & Sales Workers (ever) | 264 | 15 | 1.1 (0.6-2.0) | 1.2 (0.6-2.1) | 1.0 (0.6-1.9) | 579 | 27 | 1.3 (0.7-2.7) | 1.3 (0.7-2.6) | 1.3 (0.7-2.9) |
| 6. Agriculture & Fishery Workers (ever) | 279 | 21 | 1.2 (0.7-2.1) | 1.1 (0.6-1.9) | 1.1 (0.6-2.0) | 186 | 12 | 1.4 (0.7-2.9) | 1.4 (0.7-2.9) | 1.4 (0.7-2.9) |
| 7. Trades Workers (ever) | 324 | 18 | 0.8 (0.4-1.4) | 0.8 (0.4-1.4) | 0.8 (0.4-1.4) | 63 | 6 | 2.1 (1.1-3.9) | 2.2 (0.9-5.4) | 2.2 (1.1-5.3) |
| 8. Plant/Machine Operators & Assemblers (ever) | 420 | 30 | 1.2 (0.7-2.2) | 1.2 (0.6-2.2) | 1.2 (0.6-2.1) | 273 | 21 | 2.0 (1.1-3.9)***** | 2.0 (1.0-3.9)***** | 2.1 (1.1-4.0)***** |
| 9. Elementary Occupations (ever) | 261 | 18 | 1.2 (0.7-2.2) | 1.0 (0.6-1.9) | 1.2 (0.6-2.1) | 261 | 18 | 2.1 (1.1-4.0)***** | 2.0 (1.1-3.9)***** | 2.1 (1.1-4.0)***** |
| *****P value <0.05, ******P value <0.01. | | | | | | | | | | |
| Following IDI protocols, frequencies have been rounded to the nearest multiple of three and percentages calculated from those rounded counts. The hazard ratios and associated 95% confidence intervals are presented in their raw form and were calculated using the unrounded counts. (S = suppressed) | | | | | | | | | | |
| ^a^Participants with missing BMI or pack years information excluded: 82 female and 22 male NZWS survey participants and 122 female and 31 Māori NZWS survey. | | | | | | | | | | |
| ^b^Adjusted for age group, high deprivation and smoking status. | | | | | | | | | | |
| ^c^Adjusted for age group, high deprivation and pack years. | | | | | | | | | | |
| ^d^Adjusted for age group, high deprivation, smoking status and BMI. | | | | | | | | | | |
