# Supplementary material for: A longitudinal linkage study of occupation and ischaemic heart disease in the general and Māori populations of New Zealand
Source: PLoS One. 2022 Jan 21;17(1):e0262636. doi: 10.1371/journal.pone.0262636 (PMC8782384; doi:10.1371/journal.pone.0262636)
Supplement: S2 Table — (DOCX) [file pone.0262636.s002.docx]

| **S2 Table: Associations between NZSCO two-digit occupational groups and IHD** | | | | | | | | |
| --- | --- | --- | --- | --- | --- | --- | --- | --- |
|  | **Total**  **(n)** | **IHD cases**  **(n)** | **HR (95%CI)^a^** | **HR (95%CI)^b^** | **Total**  **(n)** | **IHD cases**  **(n)** | **HR (95%CI)^a^** | **HR (95%CI)^b^** |
| **NZWS** | **Males** | | | | **Females** | | | |
| 11. Legislators & Administrators | 30 | S | S | S | 21 | S | S | S |
| 12. Corporate Managers | 426 | 33 | 0.9 (0.6-1.4) | 1.0 (0.6-1.5) | 324 | 9 | 1.1 (0.5-2.3) | 1.1 (0.5-2.3) |
| 21. Physical, Mathematical & Engineering Science Professionals | 171 | 9 | 0.8 (0.4-1.4) | 0.8 (0.4-1.5) | 45 | S | S | S |
| 22. Life Science & Health Professionals | 48 | S | S | S | 159 | S | S | S |
| 23. Teaching Professionals | 96 | 6 | 0.9 (0.5-1.9) | 0.9 (0.5-2.0) | 270 | 9 | 1.0 (0.5-2.2) | 1.0 (0.5-2.3) |
| 24. Other Professionals | 132 | S | S | S | 195 | S | S | S |
| 31. Physical Science & Engineering Assoc. Professionals | 168 | 12 | 0.8 (0.4-1.5) | 0.8 (0.4-1.5) | 102 | S | S | S |
| 32. Life Science & Health Assoc. Professionals | 21 | S | S | S | 144 | S | S | S |
| 33. Other Assoc. Professionals | 312 | 12 | 0.4 (0.2-0.8)****** | 0.4 (0.2-0.8)****** | 468 | 9 | 0.7 (0.3-1.5) | 0.7 (0.3-1.5) |
| 41. Office Clerks | 240 | 24 | 1.5 (0.9-2.3) | 1.6 (1.0-2.5) | 663 | 18 | 0.8 (0.4-1.6) | 0.8 (0.4-1.6) |
| 42. Customer Services Clerks | 63 | 6 | 1.7 (0.8-3.6) | 1.6 (0.8-3.5) | 366 | 9 | 1.0 (0.5-2.1) | 1.0 (0.5-2.1) |
| 51. Personal & Protective Services Workers | 201 | 9 | 0.8 (0.4-1.5) | 0.7 (0.4-1.4) | 540 | 12 | 0.9 (0.5-1.9) | 0.9 (0.4-1.7) |
| 52. Salespersons, Demonstrators & Models | 222 | 9 | 0.5 (0.2-1.1) | 0.5 (0.2-1.0)***** | 411 | 18 | 2.3 (1.2-4.5)****** | 2.2 (1.2-4.3)***** |
| 61. Market-Oriented Agricultural & Fishery Workers | 315 | 24 | 1.0 (0.5-1.5) | 0.9 (0.6-1.5) | 183 | 6 | 1.2 (0.5-2.9) | 1.2 (0.5-2.8) |
| 71. Building Trades Workers | 231 | 18 | 1.2 (0.8-2.3) | 1.2 (0.5-2.8) | 12 | S | S | S |
| 72. Metal & Machinery Trades Workers | 195 | 18 | 1.4 (0.8-2.3) | 1.3 (0.8-2.1) | 6 | S | S | S |
| 73. Precision Trades Workers | 27 | S | S | S | 18 | S | S | S |
| 74. Other Craft & Related Trades Workers | 66 | 6 | 1.2 (0.5-2.8) | 1.1 (0.5-2.5) | 27 | S | S | S |
| 81. Industrial Plant Operators | 78 | S | S | S | 12 | S | S | S |
| 82. Stationary Machine Operators & Assemblers | 210 | 21 | 1.5 (0.9-2.4) | 1.3 (0.8-2.1) | 177 | 6 | 1.5 (0.7-3.4) | 1.4 (0.6-3.2) |
| 83. Drivers & Mobile Machinery Operators | 171 | 15 | 1.0 (0.6-1.7) | 0.9 (0.5-1.6) | 15 | S | S | S |
| 84. Building & Related Workers | 33 | S | S | S | S | S | S | S |
| 91. Labourers & Related Elementary Service | 327 | 27 | 1.3 (0.8-2.1) | 1.2 (0.8-1.9) | 237 | 6 | 1.2 (0.5-2.6) | 1.1 (0.5-2.5) |
| **Māori NZWS** | **Males** | | | | **Females** | | | |
| 11. Legislators & Administrators | 18 | S | S | S | 18 | S | S | S |
| 12. Corporate Managers | 192 | 15 | 1.3 (0.7-2.3) | 1.4 (0.7-2.5) | 255 | S | S | S |
| 21. Physical, Mathematical & Engineering Science Professionals | 45 | S | S | S | 27 | S | S | S |
| 22. Life Science & Health Professionals | 21 | S | S | S | 96 | S | S | S |
| 23. Teaching Professionals | 66 | 6 | 1.3 (0.5-3.0) | 1.2 (0.5-2.9) | 186 | 9 | 1.4 (0.6-2.9) | 1.4 (0.7-2.9) |
| 24. Other Professionals | 69 | S | S | S | 111 | S | S | S |
| 31. Physical Science & Engineering Assoc. Professionals | 63 | S | S | S | 42 | S | S | S |
| 32. Life Science & Health Assoc. Professionals | 18 | S | S | S | 66 | S | S | S |
| 33. Other Assoc. Professionals | 177 | 9 | 0.9 (0.5-1.9) | 0.9 (0.5-1.9) | 333 | 9 | 0.4 (0.2-1.0)***** | 0.4 (0.2-1.0)***** |
| 41. Office Clerks | 150 | 9 | 1.0 (0.5-2.0) | 1.0 (0.5-2.1) | 417 | 9 | 0.3 (0.2-0.7)****** | 0.3 (0.2-0.7)****** |
| 42. Customer Services Clerks | 39 | S | S | S | 249 | 6 | 0.6 (0.2-1.4) | 0.6 (0.2-1.3) |
| 51. Personal & Protective Services Workers | 195 | 12 | 1.3 (0.7-2.5) | 1.3 (0.7-2.4) | 528 | 24 | 1.4 (0.7-2.6) | 1.4 (0.7-2.6) |
| 52. Salespersons, Demonstrators & Models | 123 | S | S | S | 270 | 12 | 1.1 (0.5-2.2) | 1.1 (0.5-2.2) |
| 61. Market-Oriented Agricultural & Fishery Workers | 297 | 24 | 1.3 (0.7-2.2) | 1.2 (0.7-2.2) | 210 | 12 | 1.5 (0.8-3.0) | 1.5 (0.8-3.0) |
| 71. Building Trades Workers | 174 | 9 | 0.6 (0.3-1.3) | 0.6 (0.3-1.2) | 18 | S | S | S |
| 72. Metal & Machinery Trades Workers | 114 | 6 | 0.9 (0.4-1.9) | 0.9 (0.4-2.0) | 9 | S | S | S |
| 73. Precision Trades Workers | 15 | S | S | S | 18 | S | S | S |
| 74. Other Craft & Related Trades Workers | 69 | 6 | 1.9 (0.9-4.3) | 1.8 (0.8-4.1) | 24 | S | S | S |
| 81. Industrial Plant Operators | 108 | 9 | 1.0 (0.5-2.2) | 0.9 (0.4-2.1) | 24 | S | S | S |
| 82. Stationary Machine Operators & Assemblers | 285 | 24 | 1.5 (0.9-2.6) | 1.4 (0.8-2.5) | 282 | 21 | 2.4 (1.3-4.4)****** | 2.4 (1.3-4.4)****** |
| 83. Drivers & Mobile Machinery Operators | 177 | 12 | 0.9 (0.5-1.8) | 0.9 (0.5-1.8) | 36 | S | S | S |
| 84. Building & Related Workers | 36 | S | S | S | S | S | S | S |
| 91. Labourers & Related Elementary Service | 369 | 21 | 1.5 (0.8-2.6) | 1.4 (0.8-2.4) | 294 | 18 | 2.0 (1.1-3.8)***** | 2.0 (1.1-3.8)***** |
| *****P value <0.05, ******P value <0.01. | | | | | | | | |
| Following IDI protocols, frequencies have been rounded to the nearest multiple of three and percentages calculated from those rounded counts. The hazard ratios and associated 95% confidence intervals are presented in their raw form and were calculated using the unrounded counts. (S = suppressed) | | | | | | | | |
| ^a^Adjusted for age group. | | | | | | | | |
| ^b^Adjusted for age group, high deprivation and smoking status. | | | | | | | | |
