# Supplementary material for: A longitudinal linkage study of occupation and ischaemic heart disease in the general and Māori populations of New Zealand
Source: PLoS One. 2022 Jan 21;17(1):e0262636. doi: 10.1371/journal.pone.0262636 (PMC8782384; doi:10.1371/journal.pone.0262636)
Supplement: S3 Table — (DOCX) [file pone.0262636.s003.docx]

| **S3 Table: Associations between industry group and IHD** | | | | | | | | |
| --- | --- | --- | --- | --- | --- | --- | --- | --- |
| **Occupational Group** | **Total**  **(n)** | **IHD cases**  **(n)** | **HR (95%CI)^a^** | **HR (95%CI)^b^** | **Total**  **(n)** | **IHD cases**  **(n)** | **HR (95%CI)^a^** | **HR (95%CI)^b^** |
| **NZWS** | **Males** | | | | **Females** | | | |
| A. Agriculture, Forestry & Fishing (ever) | 309 | 27 | 1.0 (0.6-1.6) | 0.9 (0.6-1.5) | 201 | S | S | S |
| (employed 10+ years) | 129 | 12 | 0.8 (0.4-1.6) | 0.8 (0.4-1.6) | 51 | S | S | S |
| C. Manufacturing (ever) | 609 | 54 | 1.2 (0.8-1.8) | 1.2 (0.8-1.7) | 468 | 18 | 2.0 (1.1-3.9)***** | 1.9 (1.1-3.7)***** |
| (employed 10+ years) | 252 | 30 | 1.6 (1.0-2.5) | 1.5 (0.9-2.3) | 102 | S | S | S |
| E. Construction (ever) | 381 | 27 | 1.0 (0.6-1.5) | 0.9 (0.6-1.4) | 93 | S | S | S |
| (employed 10+ years) | 159 | 12 | 0.8 (0.4-2.3) | 0.7 (0.4-1.4) | 9 | S | S | S |
| F. Wholesale Trade (ever) | 213 | 12 | 0.8 (0.4-1.4) | 0.8 (0.5-1.5) | 165 | S | S | S |
| (employed 10+ years) | 51 | S | S | S | 9 | S | S | S |
| G. Retail Trade (ever) | 396 | 21 | 0.7 (0.4-1.2) | 0.7 (0.4-1.1) | 609 | 15 | 1.1 (0.8-2.1) | 1.0 (0.5-2.0) |
| (employed 10+ years) | 111 | 9 | 0.8 (0.4-1.7) | 0.9 (0.4-1.7) | 99 | S | S | S |
| H. Accommodation, Cafes & Restaurants (ever) | 132 | 6 | 0.9 (0.4-1.9) | 0.8 (0.4-1.8) | 333 | 9 | 1.2 (0.6-2.5) | 1.2 (0.5-2.5) |
| (employed 10+ years) | 18 | S | S | S | 30 | S | S | S |
| I. Transport & Storage (ever) | 204 | 18 | 1.1 (0.7-1.9) | 1.1 (0.7-1.8) | 126 | 6 | 2.2 (0.9-5.3) | 2.1 (0.9-5.1) |
| (employed 10+ years) | 69 | 12 | 1.9 (1.0-3.5) | 1.8 (1.0-3.4) | 18 | S | S | S |
| J. Communication Services (ever) | 102 | 9 | 1.4 (0.7-2.8) | 1.5 (0.7-2.9) | 144 | S | S | S |
| (employed 10+ years) | 24 | S | S | S | 27 | S | S | S |
| L. Property & Business Services (ever) | 324 | 21 | 0.8 (0.5-1.3) | 0.9 (0.5-1.4) | 474 | 12 | 0.8 (0.4-1.6) | 0.8 (0.4-1.7) |
| (employed 10+ years) | 93 | 6 | 0.6 (0.2-1.3) | 0.6 (0.3-1.4) | 75 | S | S | S |
| M. Government Admin. & Defence (ever) | 267 | 27 | 1.3 (0.8-2.0) | 1.3 (0.8-0.2) | 285 | 12 | 1.7 (0.8-3.4) | 1.7 (0.9-3.6) |
| (employed 10+ years) | 90 | 9 | 1.3 (0.7-2.5) | 1.4 (0.7-2.7) | 54 | S | S | S |
| N. Education (ever) | 150 | 9 | 0.9 (0.5-1.7) | 0.9 (0.5-1.7) | 447 | 12 | 0.7 (0.3-1.5) | 0.7 (0.4-1.6) |
| (employed 10+ years) | 54 | 6 | 0.9 (0.4-2.0) | 0.9 (0.4-2.1) | 141 | S | S | S |
| O- Health Communication Services (ever) | 78 | S | S | S | 561 | 15 | 1.0 (0.5-1.9) | 0.9 (0.5-1.7) |
| (employed 10+ years) | 30 | S | S | S | 198 | S | S | S |
| P. Cultural & Recreational Services (ever) | 108 | 6 | 0.8 (0.4-1.9) | 0.8 (0.4-1.9) | 180 | S | S | S |
| (employed 10+ years) | 12 | S | S | S | 24 | S | S | S |
| Q. Personal & Other Services (ever) | 129 | 12 | 1.3 (0.7-2.4) | 1.3 (0.7-2.4) | 228 | 9 | 1.3 (0.6-2.9) | 1.3 (0.6-2.8) |
| (employed 10+ years) | 33 | S | S | S | 30 | S | S | S |
| **Māori NZWS** | **Males** | | | | **Females** | | | |
| A. Agriculture, Forestry & Fishing (ever) | 288 | 24 | 1.3 (0.7-2.3) | 1.2 (0.7-2.2) | 228 | 12 | 1.4 (0.7-2.8) | 1.4 (0.7-2.8) |
| (employed 10+ years) | 84 | 9 | 1.4 (0.7-3.0) | 1.4 (0.6-2.9) | 54 | 6 | 2.6 (1.1-6.2)****** | 2.5 (1.0-6.1)***** |
| C. Manufacturing (ever) | 435 | 30 | 1.1 (0.6-1.9) | 1.0 (0.6-1.7) | 456 | 24 | 1.6 (0.8-3.1) | 1.6 (0.8-3.0) |
| (employed 10+ years) | 189 | 15 | 1.0 (0.5-2.0) | 1.0 (0.5-1.9) | 90 | 6 | 1.7 (0.6-4.3) | 1.6 (0.6-4.2) |
| E. Construction (ever) | 294 | 18 | 0.9 (0.5-1.6) | 0.9 (0.5-1.5) | 57 | S | S | S |
| (employed 10+ years) | 114 | 6 | 0.8 (0.4-1.8) | 0.8 (0.4-1.8) | 6 | S | S | S |
| G. Retail Trade (ever) | 261 | 15 | 0.8 (0.4-1.6) | 0.8 (0.4-1.6) | 468 | 12 | 0.7 (0.4-1.4) | 0.7 (0.4-1.4) |
| (employed 10+ years) | 60 | S | S | S | 63 | S | S | S |
| H. Accommodation, Cafes & Restaurants (ever) | 81 | S | S | S | 306 | 12 | 0.7 (0.4-1.5) | 0.7 (0.4-1.5) |
| (employed 10+ years) | 9 | S | S | S | 45 | S | S | S |
| I. Transport & Storage (ever) | 162 | 12 | 0.8 (0.4-1.6) | 0.8 (0.4-1.6) | 111 | S | S | S |
| (employed 10+ years) | 57 | S | S | S | 18 | S | S | S |
| J. Communication Services (ever) | 66 | S | S | S | 141 | 6 | 1.0 (0.4-2.4) | 1.0 (0.4-2.4) |
| (employed 10+ years) | 15 | S | S | S | 21 | S | S | S |
| L. Property & Business Services (ever) | 144 | 9 | 0.7 (0.3-1.6) | 0.8 (0.3-1.7) | 270 | 15 | 1.8 (1.0-3.5) | 1.9 (1.0-3.5) |
| (employed 10+ years) | 30 | S | S | S | 39 | S | S | S |
| M. Government Admin. & Defence (ever) | 210 | 18 | 1.4 (0.8-2.4) | 1.4 (0.8-2.5) | 231 | 6 | 0.6 (0.3-1.4) | 0.6 (0.3-1.4) |
| (employed 10+ years) | 69 | 6 | 1.1 (0.5-2.7) | 1.2 (0.5-3.0) | 45 | S | S | S |
| N. Education (ever) | 111 | 9 | 1.3 (0.7-2.8) | 1.3 (0.6-2.7) | 336 | 15 | 1.1 (0.6-2.2) | 1.1 (0.6-2.2) |
| (employed 10+ years) | 39 | S | S | S | 108 | 9 | 1.2 (0.5-2.7) | 1.2 (0.5-2.7) |
| O. Health Communication Services (ever) | 75 | 6 | 1.8 (0.9-3.9) | 1.9 (0.9-4.0) | 426 | 24 | 1.4 (0.8-2.7) | 1.5 (0.8-2.7) |
| (employed 10+ years) | 39 | S | S | S | 108 | 9 | 1.7 (0.8-3.7) | 1.2 (0.5-2.7) |
| Q. Personal and Other Services (ever) | 120 | 9 | 0.8 (0.4-1.8) | 0.8 (0.4-1.8) | 189 | 9 | 0.9 (0.4-2.1) | 0.9 (0.4-2.1) |
| (employed 10+ years) | 27 | S | S | S | 30 | S | S | S |
| *****P value <0.05, ******P value <0.01. | | | | | | | | |
| Following IDI protocols, frequencies have been rounded to the nearest multiple of three and percentages calculated from those rounded counts. The hazard ratios and associated 95% confidence intervals are presented in their raw form and were calculated using the unrounded counts. (S = suppressed) | | | | | | | | |
| ^a^Adjusted for age group | | | | | | | | |
| ^b^Adjusted for age group, high deprivation and smoking status | | | | | | | | |
| Industries with no participants from either sex have been excluded (NZWS: B (mining), D and K; Māori NZWS: B, D, F, K | | | | | | | | |
